# Restoration of Occlusion by the Casting Process

**Published:** 1913-06-15

**Authors:** J. Lowe Young

**Affiliations:** New York City


### RESTORATION OF OCCLUSION BY THE CASTING PROCESS.

BY DR. J. LOWE YOUNG, NEW YORK CITY.

Read before the Second District Dental Society, March, 1913, and published in the May "Items of Interest."

Considerable thought has been given to selecting a suitable title for this paper, as it is deemed advisable to discuss only what is included in the title. Consequently, we are confronted with two main questions: first, a careful study of the teeth when in occlusion; and, second, cast restorations, whether they be for inlays, crowns or bridges.

Previous to the writings of Dr. Edward H. Angle on the correction of malocclusion, the necessity of restoring normal occlusion as a result of orthodontic treatment was not recognized by the dental profession. Normal occlusion, then, became the basis of the classification and diagnosis of all cases and immediately placed orthodontia upon a scientific foundation, which fact mainly has enabled it to advance with such rapid strides during the last ten years.

For the orthodontist to correct malocclusion, he must of necessity have a clear and definite understanding of this ideal condition which he is attempting to restore. Thus it is that normal occlusion is the one supreme picture which the orthodontist has in mind, the beginning and the end of his anticipation of treatment, the ideal which governs the daily progress of correction of malocclusion, the standard in occlusal relations which above all it is desirable to obtain.

Examined analytically, this condition of normal occlusion exhibits first, normal structure of the teeth, collectively and individually; and second, normal functions of the teeth, collectively and individually. Normal structure of the teeth will imply the perfection of form of the individual teeth and of each dental arch. Normal functions will imply normal occlusal relations of the inclined planes of the cusps of the individual teeth.

With this conception of normal occlusion it will be apparant that the loss of one tooth, or of even one cusp of one tooth, or, to be more exact, the loss of any portion of the mesio-distal diameter, will to just that degree destroy both normal structure and normal function. It is also apparent to those who have seriously studied this question that it is of equal importance to properly restore the mesio-distal diameter of the *deciduous* molars where fillings have been inserted on their approximal surfaces.

Did it ever occur to you that the orthodontist often works for years to build up this normal occlusion, only to have it pulled down in a day by the ruthless extraction of a single tooth, or by the lack of restoration of cusp contour or approximal contact in making fillings or inlays?

It would seem, therefore, that the dentist must share the responsibility of the orthodontist in emphasizing the importance of normal occlusion by preserving it at all times, or at least by not destroying it.

Hence, if the general practitioner is to properly restore any part of the dental apparatus, he, like the orthodontist, must have in his minds eye the forms, surfaces and positions of the dental organs when normal.

The value of approximal contact, the proper occlusion of each cusp, the size of each fossa, the shape of each sulcus, and the direction and depth of each groove, should be known to him who aspires to restore or re-create these anatomical forms.

In this brief paper it is my purpose to call your attention especially to the one phase of normal occlusion represented in the relation of the occluding surfaces of the teeth of one dental arch to the same surfaces of the teeth of the opposing arch.

At the same time I wish to appeal to you to use the same standard of normal occlusion as a guide in your work that the orthodontist uses, so that when you examine a set of plaster models made from accurate plaster impressions you will not only consider the teeth of one arch in their mesio-distal and bucco-lingual relations to those of the opposing arch, but that you will note the position of each cusp of each tooth and its relation to the cusps of the teeth of the opposing arch; for, when carefully considered and thoroughly understood, their proper reproduction becomes of the utmost importance to the general practitioner and the prosthodontist as well as to the orthodontist.

It is my further purpose to direct your particular attention to the grooves, the pits, the ridges and the inclines found on the occluding surfaces of the teeth, and to consider the possibilities of their reproduction in your work. These features can be best studied from the natural teeth wherever it is possible to procure a skull having all the teeth in normal occlusion.

Those of you who purpose to seriously study these organs will do well to have Dr. Black's "Dental Anatomy" along with your anatomical specimens. It is really wonderful how carefully each anatomical landmark is defined by this writer. To attempt to go into detail as he has done in describing the occlusal surface of each tooth would make this paper too long, but it is deemed advisable to refer you to his Fourth Edition or Third Edition, page 81, for what he says of this surface of the lower first molar. This tooth has been chosen because of its great importance to the orthodontist in his work; and also from the fact that so many of these teeth require large restorations on their occusal surfaces previous to the eruption of the premolars and second molars.

When we consider how much more we get out of this quotation with the pictures before us on the screen, it must be apparent to everyone that in studying this work of dental anatomy we must do as Dr Black has advised and have several specimens of each tooth before us as we read the text. Great benefit may also be derived by careful consideration of plaster models, made from accurate plaster impressions, such as the orthodontist is in the habit of making. By means of such models, the relation of the cusps, fossae, sulci and ridges of the occluding surfaces of the teeth of one arch to the same surfaces of the teeth of the opposing arch can be studied from the buccal, lingual and occlusal views.

My object, therefore, in bringing this phase of occlusion to your attention has a more definite purpose than the subject as it is related to the work of the orthodontist, and yet it is of great importance to him because it bears such a close relation to his most difficult problem, namely, the retention of teeth that have been in malocclusion. Frequently the result of several years of most painstaking efforts of the orthodontist is thwarted by improper fillings and inlays.

It therefore appears that if in any way we can help each other in maintaining normal occlusion we should do so by trying to work in harmony.

Several years previous to my taking up orthodontia as a specialty, which was several years previous to Dr. Taggart's giving to the profession the casting process, a man came to my office to demonstrate the use of Watt's crystal gold. In our discussion of gold fillings generally he spoke several times of beautiful gold restorations, and I finally said to him: "Doctor, I have never seen a beautiful gold restoration." He looked at me and said: "What do you mean?" I answered him: "Just what I say." He said: "I wish you would explain yourself." So I said to him: "Are you willing to admit that for a gold restoration to be beautiful it must reproduce the lost portion of the teeth?" To this he agreed. Then I said: "In the anterior teeth the difference in color renders it improper to term the best gold fillings beautiful." "But," he said, "what about the back teeth that do not show, and where the esthetic question does not enter?" My answer was that I had never seen a gold restoration on the occlusal surface of a molar or a bicuspid that even approached the original shape of the tooth, nor did I know how to make one. Since then I have had the opportunity to see gold restorations from the hands of many of our best operators and I am of the opinion still that there are no beautiful gold fillings.

The deficiency of these fillings and inlays to which I refer is due occasionally to the failure of the operator to reproduce a cusp, but more often to his failure to reproduce the fossae, sulci, pits, grooves, ridges and the mesio-distal diameter as they are found in the natural teeth.

That it is Nature's plan to have cusps of a proper length, and fossae of a proper depth and sulci of a certain form, and ridges of a definite shape, in order to make the dental apparatus efficient, a careful study of these occlusal surfaces in their natural state will show conclusive proof.

The surgeon, to be successful, must know his anatomy.

The late J. E. Garretson, was often heard to remark to his students that before attempting the simplest surgical operation, unless in cases of extreme emergency, he always carefully read the anatomy of the part to be operated on before entering the operating room. Would it not be well for every man who attempts to make a cast-gold inlay to carefully read and study what Dr. Black says in description of the occlusal surface of the relative tooth?

Here let us note that the bottoms of the grooves, when normal, are never reached by the cusps of the teeth of the opposing jaw. In this respect the old-fashioned millstones were patterned after the grinding surfaces of the teeth, and whenever the miller allowed these stones to become dull, so that the grooves were very much reduced in depth, though not entirely obliterated, the grist was invariably spoiled. In like manner, whenever the dentist fails to reproduce the grooves, pits and ridges in restoring lost portions of the occluding surfaces of the dental organs, does he interfere with their efficiency for masticating food.

It is not the intention to give you the impression that this question applies only to teeth in normal occlusion, for if such were the case it would not be worth while imposing upon your time with such a paper. But, in order that you should be able to adapt your restorations to the best advantage in maloccluded and mutilated cases, it is of the utmost importance that you should first thoroughly understand normal occlusion. And if you find it exacting to reproduce the occlusal surfaces of the teeth in your restorations where they are in normal occlusion, I am sure it will be a greater tax for you to make proper restorations in the maloccluded and mutilated cases.

### TAGGART'S TEACHING NOT FOLLOWED.

I am aware that this paper is somewhat of a criticism on your efforts, and withal it is my desire to be fair in this matter. It is a little over five years since Dr. Wm. H.

Taggart gave to the profession his wonderful technique in this work, and it is not to be wondered at, that, in adapting yourselves to this new method of practice and solving all the intricate problems which you have undoubtedly encountered, you were quite content when the occlusal surfaces of your inlays were equal to your best efforts when gold foil was used in making these restorations.

I wish to call your attention, however, to the fact that the specimen inlays which Dr. Taggart passed around at the November, 1907, meeting, showed beautiful cusp restoration and normal depth of fossae. A porcelain-faced gold-shell crown that I have recently seen, made by him many years ago, caused me to infer that it was largely due to his desire to reproduce the normal shape of the occlusal surfaces of the teeth that he was encouraged to persevere to the point where he developed this wonderful technique which he has given to the profession.

Now, with this idea of the normal occlusal surface in mind, let us, by way of contrast, consider the meager, inefficient manner in which the average practitioner attempts to reproduce them, and see how far short he falls from the ideal in this respect.

### A CASE FROM PRACTICE.

A case that has been under my observation for a number of years for orthodontic work, and of which I have several sets of models, impressed upon me the probable mutual benefit of discussing this subject with you.

By studying the models of this case and comparing the occluding surfaces of the teeth before and after the inlays were inserted, it will be seen how lacking are the fossae, sulci, grooves, pits and ridges in these otherwise beautiful inlays. It will be observed that there are almost flat surfaces, and in many cases an over contour on the occluding surfaces. In several places the cusp of a tooth in one jaw strikes too hard on the inlay in the opposing jaw. Indeed, it is a wonder that more teeth are not split where fillings and inlays are left in such a condition.

These inlays were made by as conscientious an operator as I have ever known. I am satisfied that he would have done just such work as this for his own child and felt proud of it. The child complained of not being able to masticate food as well as she formerly could. I have seen plaster models of many cases, though less marked, showing these same defects.

The question then naturally arises how to reproduce these anatomical landmarks found on the occluding surfaces of the teeth, so as not to interfere with their efficiency, when restoring portions, or all, of their occluding surfaces.

Previous to the introduction of cast-gold inlays it seemed almost impossible, and very improbable, that this would ever be done. With the perfection of this process, it appears to be quite within the range of the careful, conscientious operator to so reproduce such anatomical landmarks in restoring any portion, or all, of the occlusal surface of a tooth, that detection of these restorations will be almost impossible, when examining plaster models made from accurate plaster impressions of such teeth.

Fearing that you will accuse me of criticising your efforts without offering any solution to the problems involved, I am compelled to offer some suggestions as a remedy for these evils, and in doing so I am venturing on new ground, as I have never had any practical experience in making cast-gold inlays.

I am not very familiar with the technique of this work, but I understand there are two methods employed, namely, the direct, and the impression method, but in either case *the occluding surface of the inlay should be made so as to accurately reproduce the natural shape of the tooth that it is to occupy, before it is cemented in place.*

By referring to the pictures shown in the quotation from Dr. Black you will observe that this necessitates the presence of cusps, fossae, grooves, pits and ridges. That it is possible to reproduce these in wax and that the same can be cast without obliterating the effects of this handiwork, will, I think, be apparent to all.

One of the great difficulties experienced by the orthodontist is to retain the mesio-distal relation after it has been established. Very frequently this trouble is due to improper fillings, or inlays, on the occluding surfaces of the teeth, particularly those of the lower first molars. If these restorations can be made so as to accurately reproduce the original shapes of these teeth, and thus permit the large mesio-lingual cusp of the upper first molar to properly seat itself each time the teeth are closed, do you not see what a powerful influence is exerted by the action of the inclined planes of this cusp on the inclined planes of the five cusps of the lower first molar, to prevent a return to a mesial or distal malocclusion, and do you not see that to a proportionate degree each reproduction of the normal occlusal surface of a tooth exerts a like helpful influence? Where all restorations accurately reproduce the original anatomical landmarks, the orthodontist will experience much less difficulty in the retention of such cases.

Granting, then, that these occlusal restorations are possible, does it not appeal to you that they are necessary from the standpoint of beauty, perfection of anatomical contour, and especially for efficiency?

In presenting to you the keen appreciation of the orthodontist, of the importance of normal occlusion it is with the hope of arousing a like appreciation in the dentist, so that in all his efforts at restoration of the lost parts of the dental apparatus he will be inspired to accurately reproduce, in the minutest detail, their anatomical shape. If I have succeeded in doing this I shall feel well repaid and will look forward with keen interest to a heartier co-operation between the dentist and the orthodontist, in the attainment of normal occlusion.—May, *Items of Interest.*

### DISCUSSION OF DR. YOUNG'S PAPER BY DR. OTTOLENGUI.

We have seen that the study of occlusion has produced great advance in two branches of dentistry, prosthodontia and orthodontia, and we are now to consider to what extent similar study will aid us in the filling of teeth. At this juncture I wish to call attention to the different relation which this study of occlusion must have upon the three branches of our profession.

The prosthodontist should comprehend occlusion, because he is called upon to replace lost organs by artificial substitutes. In the accomplishment of his aim he has the advantage, that he may handle the individual teeth as units. He may place them as he pleases and carve them to conform to his ideas. With these advantages, and possessed of a full knowledge of occlusion, the skilled prosthodontist should be capable of so articulating his artificial teeth that he may restore normal occlusion for any edentulous mouth. In this statement I am not belittling the difficulties of producing teeth that will be perfectly satisfactory in all ways. I am alluding solely to the problem of restoring occlusion with artificial teeth.

The orthodontist is more handicapped than is the prosthodontist. His province is to move teeth, but he cannot move them as does the prosthodontist. He cannot place them where he pleases, nor can he elevate or depress them as he pleases, to conform with his idea of occlusal plane and normal arch form, without encountering and overcoming great obstacles. Above all, he must deal with his teeth as he finds them. He cannot or should not alter the forms of the teeth by so much as a decimal part of a millimeter.

The proposition of the essayist that the operative dentist should restore occlusion with cast gold inlays, a totally new phase of the art is met. The general practitioner, for example, cannot and indeed is not expected to correct malocclusions merely by the filling of teeth. On the other hand, the essayist very forcefully contends that he should not produce, nor reinduce malocclusion, by his faulty methods of filling, whether with inlay, foil, or plastic.

The operative dentist should thoroughly comprehend what normal occlusion is and he should comprehend what normal occlusion does; what it does for the individual fortunate enough to possess it. A full comprehension must include the useful purpose served by the overbite in the incisive region; the reason why the cuspids have pointed crowns, and the longest and stoutest roots; the reason for the shape and proportionate sizes of the bicuspids, and why the lower bicuspids differ from the upper; the reason for the shape and position of the cusps, fossae, sulci, and ridges of the molars; the reason why the upper arch should extend beyond the lower, when in contact both bucally and incisively. To the man who is too lazy to study out all these "why's" it may be said that at least he should admit that these curious phases of the human denture exist; that existing they must serve a good purpose; and that any deviation from the normal must proportionately lessen the usefulness of the denture.

Every practitioner should thoroughly understand the why and the wherefore of normal occlusion, because the man who fills teeth is often dealing with some aberration from normal occlusion, and a knowledge of the normal is needed that he may intelligently deal with the abnormal. Still, it is only fair to the general pratitioner to call the attention of the essayist to the fact, that however ideal his preachment may be and however righteous his tenets, the man filling teeth will meet obstacles which will at times prevent the achievement of the truly ideal, for he must deal not alone with teeth in normal occlusion, but with teeth in malocclusion; likewise with occlusions disturbed by mutilation through extraction; occlusions changed on account of neglected caries, and elongation of antagonizing teeth, as well as the drifting of adjacent teeth; with occlusions altered by the malformations of individual members, and by congenital absences. And perhaps worse than all, with dentures already injured by fillings improperly formed. At this point it may not be unwise to recall a recommendation made by Dr. Ash on this floor this winter, and that is, that where the usefulness of a natural denture has been lessened or destroyed by improper fillings, the dentist might well copy the orthodontist, make study models, and recommend to the patient the removal of all such poor fillings, the same to be replaced by inlays that will restore the teeth to their highest possibilities.

At first soft gold was used, and admirably served the purpose of saving teeth. Saving the tooth was about the limit of its usefulness. With the advent of cohesive foil arrived the demand for restoration of contour, and especially of contact points for the protection of the interproximal space and the soft tissues embraced therein, as well as for the retention of the tooth in its proper place. With cohesive foil the contourists of the past became wonderfully expert in restoring the anterior teeth, and the approximal portions of the posterior teeth. But with the advent of the cast gold inlay, and especially in view of the standards set up to-night, which include a demand for restoration of cusp and sulcus, fossa and ridge, with absolute accuracy, we see how deficient has been the gold foil worker in restoring the most important part of the masticatory apparatus, the occlusal surfaces of bicuspids and molars.

We are compelled to confess that the standard set to-night, not only never has been reached with the foil filling, but that the obstacles are such that it is well nigh impossible, because it involves the carving of the cusps and sulci, out of the hard gold, in the patient's mouth. But a greater difficulty lies in the fact that whereas the pattern for the cast inlay being made of wax, the carving may be done with pointed carving tools, when working on the foil filling, rotary cutting instruments must be used, and with these quite different results are obtained. I happen to be quite capable of testifying to this, because since Dr. Young first called my attention to this matter of faulty restoration, I have been so dissatisfied with many inlays previously put in by myself that in many instances I have had the patient give me a full sitting that I might attempt to improve the occlusal surfaces of molar and bicuspid inlays, and, indeed,

I have greatly improved such as I have done, but at the same time I have learned the impossibility of getting in this way results that can be easily obtained with the cast gold inlay. Yet this would be the procedure necessary if attempting to produce true occlusal restorations with gold foil. Hence it must follow that just as we admit the need for such occlusal restorations, we must abandon the foil filling.

Dr. Young has made an appeal for the restoration of occlusion when filling teeth, and he has demonstrated the possible restoration of tooth form, but he franlky admits that he has had no practical experience in making gold inlays, and consequently he has not been able to supply us with a technique for actually restoring the occlusion in practical cases. He has simply carved inlays out of the mouth, restoring tooth form. As it happens this is exactly the method which I pursue in the mouth. For, as I have the rubber dam in place whenever it is possible to have it, and as I carve the inlay wax directly in the cavity, I have no guide whatever but a general knowledge of tooth forms, and it is not strange therefore that not infrequently I guess wrong, and so find my inlay, when cast, too high for the occlusion, however pretty the carving may be. Some say they take a "mash bite," before carving the wax. This has not been satisfactory to me. Others carve the inlay, and then try in the wax, allowing the patient to close the bite. In this way I have a few times spoiled in one moment an inlay that I had spent thirty minutes in carving. In any event, it is a risk to the margins to try a wax pattern in the mouth and have the patient *grind* upon it, and a mere *closure* does not disclose the interference which may develop under a free movement of the mandible. There are various methods suggested for obtaining the true occlusal bite, but I need not discuss them here. It is my belief that with a thorough knowledge of normal tooth forms, and of abraded tooth forms, and with practice in carving, one may become so dexterous in carving the wax that when the finished inlay is seated, it will not be too high. In any event, these high points should develop on the cusps, and consequently, if the sulci and grooves have been made deep enough, the correction of the inlay may be made after the gold is cemented to place, by using a carbon paper and grinding with the *tiniest* of carborundum stones. Even if it is not as artistic as the beautiful specimens shown by Dr. Young, inlays made in this manner will be far more useful, and nearer to true form and occlusion than are most of the inlays, or any of the foil fillings now being made.

Dr. Young has said that after carving the wax and reproducing it in gold it would be better to set the inlay as it comes from the mold rather than to mutilate it in the polishing process, and with this I agree. But if the carved wax be polished with oil of cajaput, and then properly invested and cast, no finishing will be required that cannot be done with burnishers. For this I use tantulum burnishers, but since adopting Dr. Young's suggestion as to making deep and sharp-pointed sulci, I have been obliged to reshape one tantulum burnisher for the special purpose of burnishing the sulci. It is practically a hatchet with a dull edge.

In the past we have contoured teeth, but we have not contoured them to the extent implied by the word "restore." The contourists have replaced corners, have built up incisal edges with fair accuracy, and they have even rounded out the approximal or circumferential shapes of teeth. But they have woefully failed to even fairly well imitate the occlusal surfaces of bicuspids and molars. Perhaps never until now has the dental world been fully aroused as to the importance of restoring occlusion. The orthodontist and the prosthodontist have far outstripped the dentist in this respect, but the time has now arrived when the dentist must awaken and grasp the need of making occlusion the fundamental principle of this every-day work.

Any child would tell us "teeth are made to eat with." The great Master so formed these organs that they would not only "eat," but "eat" well. The slightest loss of any part of the masticating surface of a tooth, the most minute change in its designed form, diminishes its usefulness as a masticatory organ to just that extent. Reversely, where a part or all of the occlusal surface of a tooth is lost through caries, the more nearly the dentist succeeds in restoring its original form, the more perfect does he make the patient's ability to masticate his food.

Hundred of articles have been written upon the need of restoring approximal contact as a protection to the gingiva which normally fills the interproximal space. But a close study of the anatomy of the region will demonstrate that the contact of adjacent teeth is only one of Nature's measures for the protection of the gingiva, and not the most important. At the approximal margin of the occlusal surface of molar or bicuspid will be found a marginal ridge with its most slanting plane extending toward the center of that surface, and therefore away from the approximal space. These marginal ridges lead down into gutters, which in turn carry the escaping food lingually and buccally, so that in the normal state the contact points need be but small rounded surfaces, as they are, to prevent food from crowding down against the sensitive and easily injured gingiva. Thus the absolute copying of Nature in the restoration of occlusal surfaces of molars and bicuspids not only increases the masticatory efficiency by supplying cusps and fossae, but protects the soft parts and preserves them in a state of health because of the marginal ridges and sulci.

We urge our readers, therefore to carefully study Dr. Young's paper and to apply themselves to the prompt acquirement of the technique of cast gold restorations, for the day is dawning when patients will demand that the dentist as well as the prosthodontist and the orthodontist shall make his highest aim the restoration of occlusion to as nearly normal as may be. This cannot be done with gold foil. Thus cohesive foil will be superseded by the inlay, just as it took the place of soft foil, because the American dentist will never be content with less than the highest standard of achievement.—May *Items of Interest.*

